# Dihydropyrimidinase-like 3 facilitates malignant behavior of gastric cancer

**DOI:** 10.1186/s13046-014-0066-9

**Published:** 2014-08-06

**Authors:** Mitsuro Kanda, Shuji Nomoto, Hisaharu Oya, Dai Shimizu, Hideki Takami, Soki Hibino, Ryoji Hashimoto, Daisuke Kobayashi, Chie Tanaka, Suguru Yamada, Tsutomu Fujii, Goro Nakayama, Hiroyuki Sugimoto, Masahiko Koike, Michitaka Fujiwara, Yasuhiro Kodera

**Affiliations:** 1 Department of Gastroenterological Surgery (Surgery II), Nagoya University Graduate School of Medicine, 65 Tsurumai-cho, Showa-ku, Nagoya 466-8550, Japan

**Keywords:** Gastric cancer, Dihydropyrimidinase-like 3, Expression, Prognosis, Biomarker

## Abstract

**Background:**

Gastric cancer (GC) remains to have a poor prognosis via diverse process of cancer progression. Dihydropyrimidinase-like 3 (*DPYSL3*) is a cell adhesion molecule that has been reported to be involved in the metastatic process of tumor cells. The aim of this study was to identify a novel clinically-relevant biomarker of GC.

**Methods:**

Expression analysis of *DPYSL3* mRNA and protein levels was conducted using GC cell lines and 238 pairs of surgically resected gastric tissues. Correlations between expression status of *DPYSL3* and clinicopathological parameters were investigated.

**Results:**

*DPYSL3* mRNA expression levels positively correlated with those of potentially interacting genes (*VEGF*, *FAK* and *EZR*) in GC cell lines. GC tissues from tumors with distant metastases (stage IV cancer) showed elevated expression levels of *DPYSL3* mRNA. The *DPYSL3* staining intensity in immunochemical staining was consistent with the mRNA expression patterns in GC tissues. High *DPYSL3* mRNA expression in GCs was significantly associated with more malignant phenotypes and was an independent prognostic factor. Moreover, patients with high *DPYSL3* mRNA expression had a significantly shorter recurrence free survival after curative resection. In subgroup analysis based on tumor histology, similar tendency was observed between patients with differentiated and undifferentiated GCs.

**Conclusions:**

Expression status of *DPYSL3* in GC tissues may represent a promising biomarker for the malignant behavior of GC.

## Background

Gastric cancer (GC) is the second leading cause of global cancer mortality, accounting for 700,000 deaths annually [1],[2]. More than 70% of countries worldwide have a mortality-to-incidence ratio greater than 0.8, suggesting that prevention of late presentation and modified treatment strategies are required to improve clinical outcomes [3]. In particular, distant metastases including peritoneal dissemination have been recognized as important prognostic determinants for GC patients [4],[5]. Identifying genes relevant to the malignant behavior of GC could aid clinicians in tailoring treatments by identifying high-risk patients and proposing novel molecular targets [6].

Recently, technological advances such as microarrays and next-generation sequencing have allowed for the exhaustive genomic characterization of malignancies, enhancing our understanding of cancer initiation and progression [7]-[9]. With these techniques, numerous genetic and epigenetic alterations relevant to gastric carcinogenesis and GC progression have been reported [10]. However, understanding the clinical significance of individual genes remains insufficient, despite the accumulating array data.

Dihydropyrimidinase-like 3 (*DPYSL3*) is a cell-adhesion molecule [11],[12] and actively expressed in normal tissues of cardiac myocytes, brain, pineal body, retina and smooth muscle, and moderately expressed in various tissues including gastric tissues [13]. *DPYSL3* has been reported to be involved in the metastatic process of tumor cells [14],[15]. Gao et al. conducted expression and functional analyses of *DPYSL3* in prostate cancer and found that *DPYSL3* is a metastasis suppressor that is inversely associated with the expression of vascular endothelial growth factor (VEGF) [14]. In contrast, Kawahara et al. reported that *DPYSL3* facilitates pancreatic cancer cell metastasis via a strong interaction with other cell adhesion factors, including ezrin (EZR), focal adhesion kinase (FAK) and c-SRC [15]. Thus, *DPYSL3* has attracted attention as a metastatic modulator; however, the role of *DPYSL3* expression in GC initiation and progression has not been investigated.

Here, we focused on *DPYSL3* as a potential facilitator of malignant behavior in GC. The aim of this study was to evaluate the clinical significance of *DPYSL3* expression in GC.

## Material and methods

### Ethics

This study conformed to the ethical guidelines of the World Medical Association Declaration of Helsinki‐Ethical Principles for Medical Research Involving Human Subjects and has been approved by the Institutional Review Board of Nagoya University, Japan. Written informed consent for usage of clinical samples and data, as required by the institutional review board, was obtained from all patients.

### Sample collection

Ten GC cell lines (H111, KATOIII, MKN1, MKN28, MKN45, MKN74, NUGC2, NUGC3, NUGC4 and SC-6-LCK) were obtained from the American Type Culture Collection (Manassas, VA, USA) or Tohoku University, Japan and cultured in RPMI-1640 medium supplemented with 10% fetal bovine serum in 5% CO_2_ at 37°C. Primary GC tissues and corresponding normal adjacent tissues were collected from 238 patients undergoing gastric resection for GC without neoadjuvant therapy at Nagoya University Hospital between 2001 and 2012. The collected tissue samples were immediately flash-frozen in liquid nitrogen and stored at −80°C until RNA extraction. Approximately 5 mm^2^ was extracted from each tumor sample, avoiding necrotic tissue by gross observation and only samples confirmed to comprise more than 80% tumor components by H&E staining were included in this study. Corresponding normal adjacent gastric mucosa samples were obtained from the same patient and were collected > 5 cm from the tumor edge [16]. The specimens were classified histologically using the 7th edition of the Union for International Cancer Control (UICC) classification [17]. To evaluate whether the expression status of *DPYSL3* differed by tumor histology, patients were categorized into two histological subtypes; differentiated (papillary, well differentiated and moderately differentiated adenocarcinoma) and undifferentiated (poorly differentiated adenocarcinoma, signet ring cell and mucinous carcinoma) tumors. Since 2006, adjuvant chemotherapy using S-1 (an oral fluorinated pyrimidine) has been applied to all UICC stage II–IV GC patients unless contraindicated by the patient’s condition [18],[19].

### Expression analysis of *DPYSL3* mRNA

*DPYSL3* mRNA expression levels of 10 GC cell lines and 238 primary GC tissues and corresponding normal adjacent tissues were analyzed through quantitative real-time reverse-transcription polymerase chain reaction (qRT-PCR) with an ABI StepOnePlus Real-Time PCR System (Perkin-Elmer, Applied Biosystems) as described previously [20],[21]. The primer sequences used in this study are listed in Additional file 1: Table S1. In clinical samples, mean expression level of *DPYSL3* mRNA were compared between GC tissues and corresponding normal adjacent tissues. Additionally, expression level of *DPYSL3* mRNA in GCs was compared with each patient subgroup based on UICC stage to investigate the oncological role of *DPYSL3*.

### Expression of genes potentially interacting with *DPYSL3*

The expression status of *VEGF*, *EZR*, *FAK* and *c-SRC*, which have previously been reported to be genes that potentially interact with *DPYSL3*[14],[15], was evaluated in GC cell lines through qRT-PCR. Primers specific for *VEGF*, *EZR*, *FAK* and *c-SRC* are listed in Additional file 1: Table S1.

### Immunochemical staining

*DPYSL3* protein localization was determined by immunochemical staining using 54 representative formalin-fixed and paraffin-embedded sections of well-preserved GC tissue as described previously [22],[23] with a mouse monoclonal antibody against *DPYSL3* (LS-C133161, LifeSpan BioSciences, Seattle, WA, USA) diluted 1:150 in antibody diluent (Dako, Glostrup, Denmark). Staining patterns were compared between GCs and the corresponding normal adjacent tissues, and the intensity of *DPYSL3* protein expression was graded depending on the percentage of stained cells as follows: no staining, minimal (<20%); focal (20 – 60%); and diffuse (>60%) [24],[25]. To avoid subjectivity, the specimens were randomized and coded before analysis by two independent observers blinded to the status of the samples. Each observer evaluated all specimens at least twice to minimize intra-observer variation [26].

### Evaluation of clinical significance of *DPYSL3* expression

Patients were stratified into two groups divided by the median value of *DPYSL3* mRNA expression level in cancerous tissues of the all analyzed patients; high *DPYSL3* expression (higher than the median value) and low *DPYSL3* expression (the median value or lower). Correlations between the pattern of *DPYSL3* mRNA expression and clinicopathological parameters were evaluated. Outcome analyses including disease specific survival rate, recurrence free survival rate and multivariate analysis were performed in 169 patients who underwent curative surgery (i.e. stage I - III). Additionally, the prognostic impact of *DPYSL3* mRNA expression was assessed in each patient subgroup based on tumor differentiation.

### Statistical analyses

The relative mRNA expression levels (*DPYSL3/GAPDH*) between the two groups were analyzed using the Mann–Whitney U test. The strength of a correlation between two variables was assessed by the Spearman's rank correlation coefficient. The χ^2^ test was used to analyze the association between the expression status of *DPYSL3* and clinicopathological parameters. Disease specific and recurrence free survival rates were calculated using the Kaplan–Meier method, and the difference in survival curves was analyzed using the log-rank test. We performed multivariable regression analysis to detect prognostic factors using the Cox proportional hazards model, and variables with a *P* value of < 0.05 were entered into the final model. All statistical analyses were performed using JMP 10 software (SAS Institute Inc., Cary, NC, USA). *P* < 0.05 was considered significant.

## Results

### Expression of *DPYSL3* and potentially interacting genes in GC cell lines

**Figure 1:**
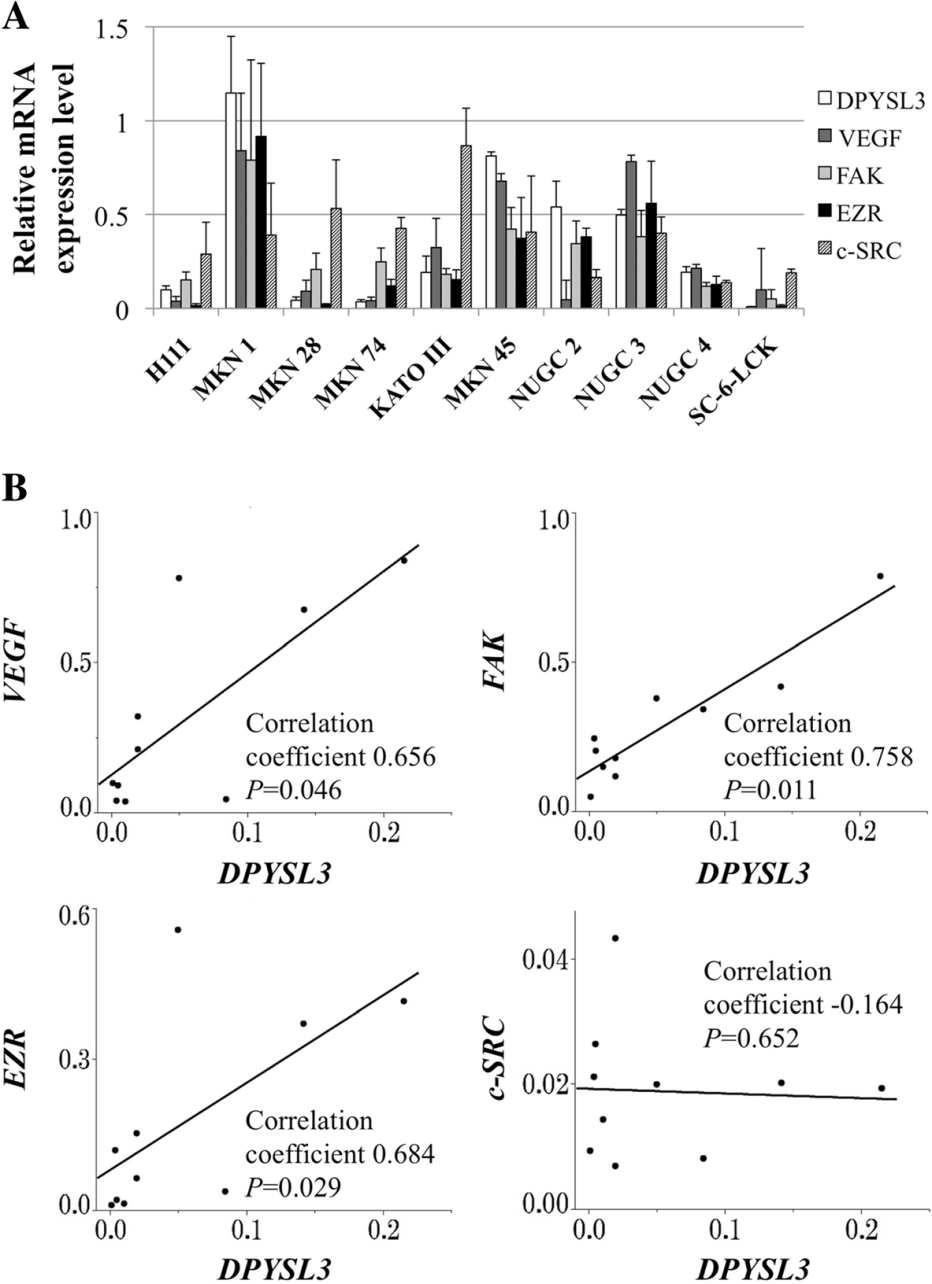
**Expression profile of GC cell lines. (A)** Expression status of *DPYSL3* and potentially interacting genes in GC cell lines. Differential mRNA expression in GC cell lines was observed. Error bars indicated standard deviation among three biological replicates. **(B)** Correlative analysis between the mRNA expression levels of *DPYSL3* and those of *VEGF*, *FAK*, *EZR* and *c-SRC*.

The relative mRNA expression levels of *DPYSL3* and its potential interacting genes in GC cell lines are shown in Figure 1A. There were large differences in mRNA expression level of *DPYSL3* and other genes among GC cell lines. *DPYSL3* expression levels positively correlated with those of *VEGF*, *FAK* and *EZR*, while no interaction was observed with *c-SRC* (Figure 1B).

### Patient characteristics

The patient ages ranged from 20 to 84 years (65.3 ± 11.7 years, mean ± standard deviation), and the male:female ratio was 179:59. Pathologically, 139 patients were diagnosed with undifferentiated GC and 99 with differentiated GC. According to the 7th edition of the UICC classification, 58, 40, 71 and 69 patients were in stages I, II, III and IV, respectively. Sixty of the 69 stage IV patients were diagnosed as stage IV due to positive peritoneal lavage cytology, localized peritoneal metastasis or distant lymph node metastasis including para-aortic lymph nodes. Eight patients in stage IV had synchronous liver metastasis one had lung metastasis, and they underwent gastrectomy with the purpose of controlling tumor bleeding or obstruction to the passage of food.

### Expression status of *DPYSL3* mRNA in 238 clinical GC samples

**Figure 2:**
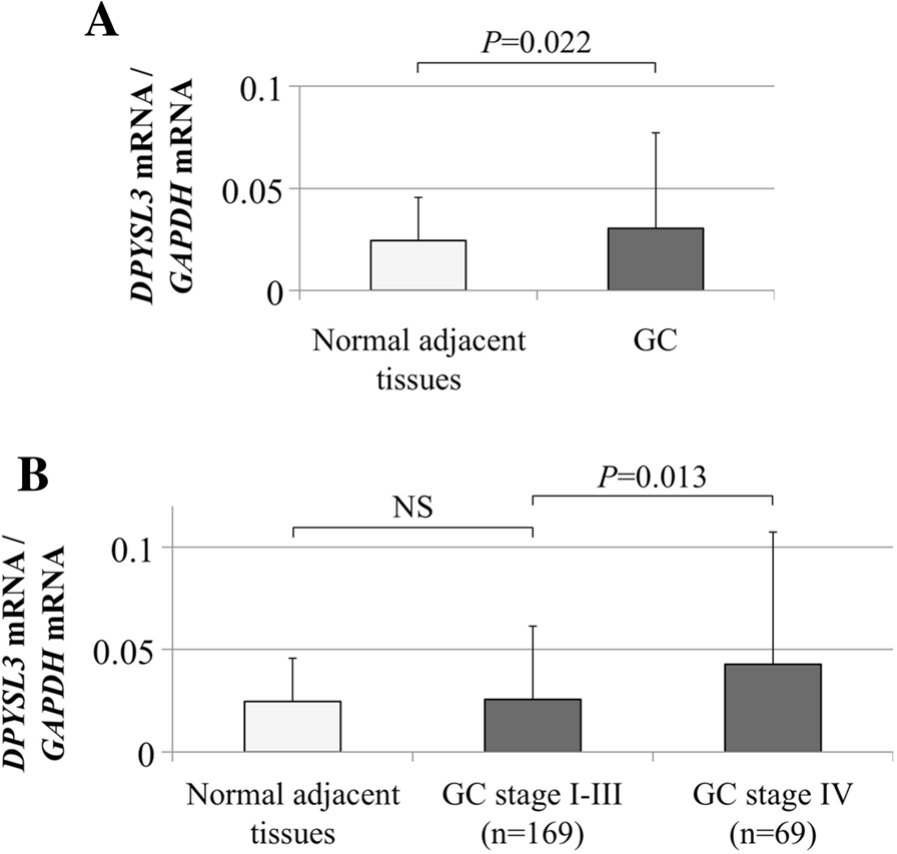
**Expression status of*DPYSL3*in clinical specimens. (A)** GC tissues showed higher mean expression levels of *DPYSL3* mRNA than corresponding normal adjacent tissues. **(B)** After subdividing patients according to UICC staging, GC tissues from patients with stage IV GC showed the highest *DPYSL3* mRNA expression levels compared with corresponding normal adjacent tissues and those from patients with stage I-III GC. NS, not significant.

Elevation of the mean expression level of *DPYSL3* mRNA was observed in GC tissues compared with the corresponding normal adjacent tissues (Figure 2A). When subdividing patients by UICC stage, *DPYSL3* expression levels were significantly higher in stage IV patients than in stage I-III patients, indicating that *DPYSL3* may promote distant metastasis (Figure 2B).

### Detection of *DPYSL3* protein

**Figure 3:**
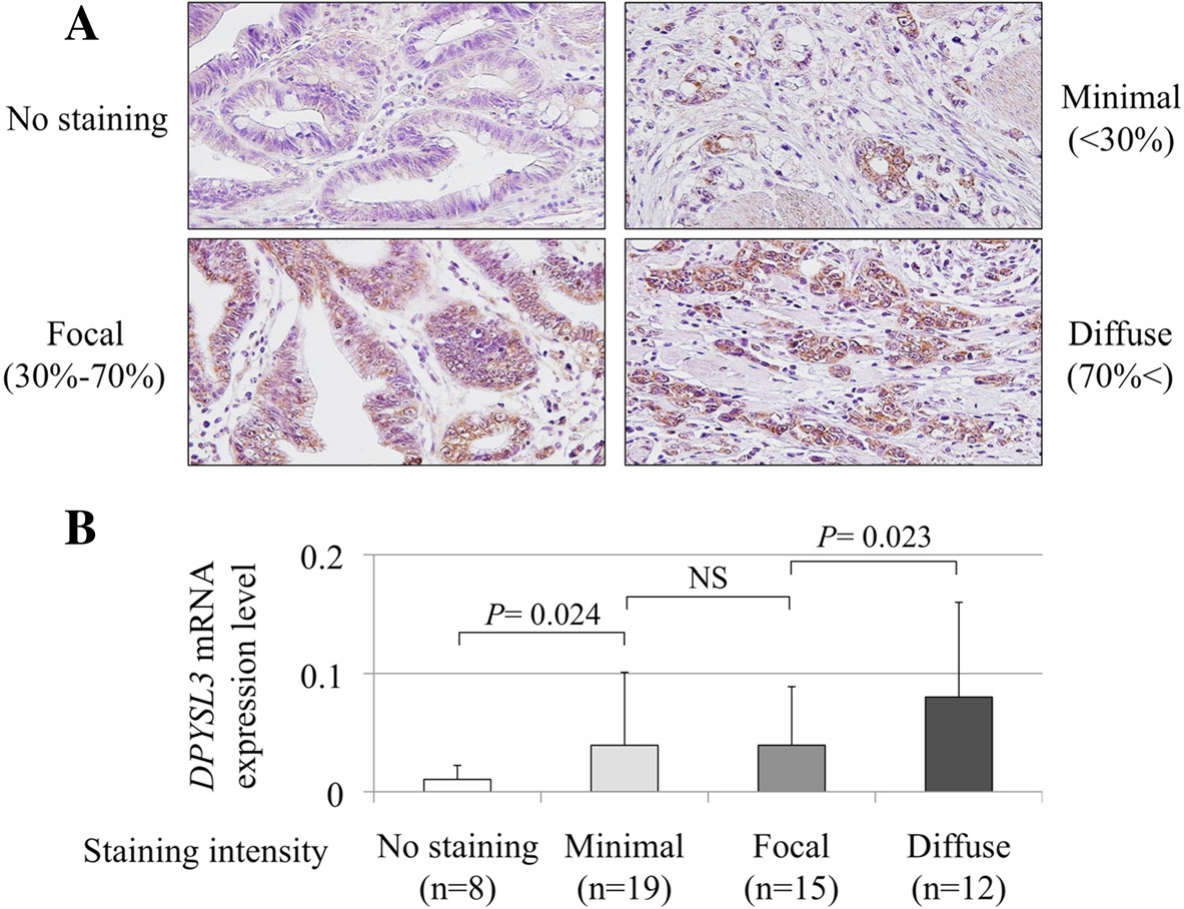
**Detection of*DPYSL3*protein. (A)** Representative cases of each *DPYSL3* staining intensity; no staining, minimal, focal and diffuse (400× magnification). **(B)** A positive correlation was observed between the expression level of *DPYSL3* mRNA and the staining intensity in GC tissues.

Representative cases with each staining grade in GC tissues are shown in Figure 3A. Diffuse staining of *DPYSL3* protein in the cytoplasm of cancerous cells was observed, whereas cells in the adjacent normal adjacent tissue had less staining. Generally, the expression patterns of *DPYSL3* protein detected by IHC were consistent with the qRT-PCR data. When grading the staining intensity of the cancerous cells, patient numbers 8, 19, 15 and 12 were categorized as no staining, minimal, focal and diffuse, respectively. A positive correlatin between the *DPYSL3* staining grade and mRNA expression levels in GC tissues was confirmed (Figure 3B).

### Prognostic impact of expression status of *DPYSL3* in gastric tissues

Correlations between expression status of *DPYSL3* mRNA and clinicopathological parameters were evaluated in 238 patients with GC. High expression level of *DPYSL3* mRNA in GCs was significantly associated with more aggressive phenotype including pT4, invasive growth, lymph node metastasis, positive peritoneal lavage cytology, and UICC stage IV, and but not tumor location (Table 1).

**Table 1:** **Association between expression level of** ***DPYSL3*** **mRNA and clinicopathological parameters in 238 patients**

**Variables**	**High** ***DPYSL3*** **mRNA in GC tissue (n)**	**Low** ***DPYSL3*** **mRNA in GC tissue (n)**	***P*-value**
Age			0.793
< 65 year	51	49	
≥ 65 year	68	70	
Gender			0.453
Male	87	92	
Female	32	27	
Carcinoembryonic antigen (ng/ml)			0.415
≤ 5	93	98	
> 5	26	21	
Carbohydrate antigen 19–9 (IU/ml)			0.504
≤ 37	95	99	
> 37	24	20	
Tumor location			0.769
Entire	12	8	
Upper third	24	27	
Middle third	37	35	
Lower third	46	49	
Tumor size (mm)			0.090
< 50	48	61	
≥ 50	71	58	
Tumor depth (UICC)			<0.001*
pT1-3	51	77	
pT4	68	42	
Histology			0.098
Papillary	1	1	
Well differentiated	4	10	
Moderately differentiated	33	48	
Poorly differentiated	74	56	
Signet ring cell	5	2	
Mucinous	2	2	
Differentiation			0.006*
Differentiated	39	60	
Undifferentiated	80	59	
Lymphatic involvement			0.016*
Absent	11	24	
Present	108	95	
Vessel invasion			0.036*
Absent	44	60	
Present	75	59	
Infiltrative growth type			<0.001*
Invasive growth	55	28	
Expansive growth	64	90	
Lymph node metastasis			<0.001*
Absent	32	57	
Present	87	62	
Peritoneal lavage cytology			0.001*
Negative	84	104	
Positive	35	15	
UICC stage			0.032*
I - III	77	92	
IV	42	27	

*Abbreviations*: *UICC* Union for International Cancer Control. *Statistically significant (*P* < 0.05).

**Figure 4:**
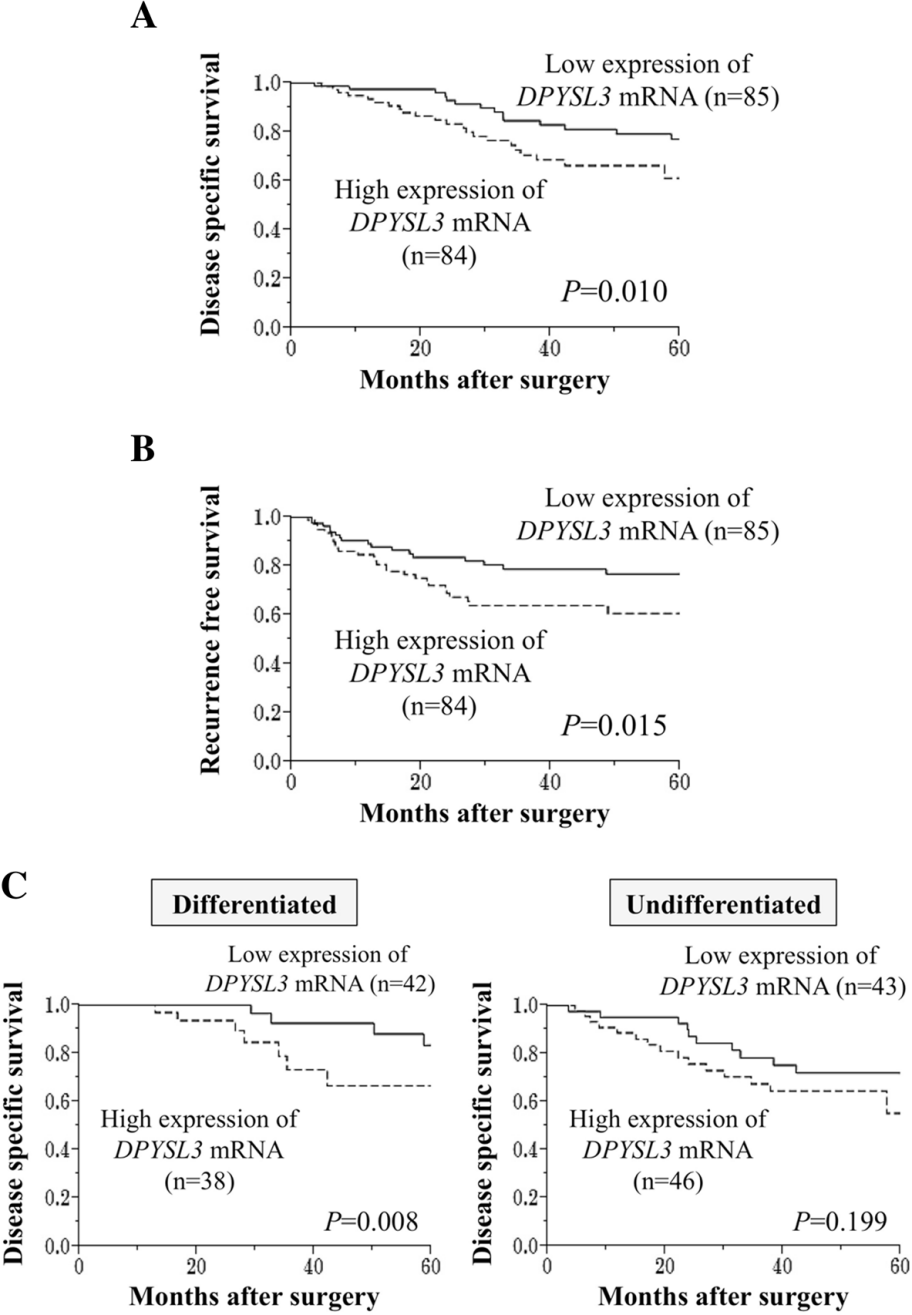
**Prognostic impact of*DPYSL3*mRNA expression in GC patients. (A)** The high *DPYSL3* mRNA expression group had significantly shorter disease specific survival than the low expression group. **(B)** Recurrence free survival was significantly shortened in the high *DPYSL3* mRNA expression group. **(C)** Curves comparing the disease specific survival of patient groups categorized by expression level of *DPYSL3* mRNA in each tumor differentiation.

Next, outcome analysis was carried out for 169 patients who underwent curative surgery. Patients with high expression level of *DPYSL3* mRNA in GCs (n = 84) were more likely to have a shorter disease specific survival than those with low expression level of *DPYSL3* mRNA (n = 85; the 5-year survival rates were 61% and 77%, respectively, *P* = 0.010; Figure 4A). Multivariate analysis identified high expression level of *DPYSL3* mRNA in GCs as an independent prognostic factor (Table 2). Moreover, high expression level of *DPYSL3* mRNA in GCs was significantly associated with shortened recurrence free survival (the 2-year survival rates were 67% in high expression group and 84% in low expression group, respectively, *P* = 0.015; Figure 4B).

**Table 2:** Prognostic factors for disease specific survival in 169 patients who underwent curative surgery

**Variable**	**n**	**Univariate**			**Multivariate**		
		**Hazard ratio**	**95% CI**	***P*-value**	**Hazard ratio**	**95% CI**	***P*-value**
Age (≥65)	97	1.38	0.73 – 2.70	0.327			
Gender (male)	128	1.27	0.60 – 2.49	0.517			
Tumor location (distal)	107	0.42	0.22 – 0.78	0.006	0.53	0.27 – 1.05	0.067
Carcinoembryonic antigen (>5 ng/ml)	27	1.71	0.73 – 3.56	0.202			
Carbohydrate antigen 19–9 (>37 IU/ml)	23	2.33	0.99 – 4.90	0.054			
Tumor size (≥50 mm)	76	3.02	1.54 – 6.35	0.001	2.06	0.98 – 4.57	0.056
Tumor depth (pT4, UICC)	55	2.82	1.50 – 5.39	0.001	1.09	0.52 – 2.32	0.815
Tumor differentiation (undifferentiated)	89	1.79	0.93 – 3.60	0.081			
Lymphatic involvement	137	5.70	1.74 – 35.2	0.002	1.12	0.14 – 6.12	0.905
Vessel invasion	83	4.10	2.02 – 9.20	<0.001	2.93	1.31 – 7.52	0.008*
Invasive growth	41	2.51	1.31 – 4.73	0.006	1.39	0.64 – 3.00	0.404
Lymph node metastasis	86	8.70	3.71 – 25.5	<0.001	4.01	1.40 – 14.6	0.008*
Expression of *DPYSL-3* mRNA (high)	84	2.36	1.22 – 4.72	0.010	2.22	1.14 – 4.49	0.019*

*Statistically significant in multivariable analysis. GC, gastric cancer; CI, confidence interval; UICC, Union for International Cancer Control.

### Subgroup analysis based on tumor differentiation

The prognostic impact of *DPYSL3* expression was evaluated in each patients subgroups classified by tumor differentiation. Although statistically significant difference was exhibited only in patients with differentiated GCs, similar tendency was observed between survival curves of patients with differentiated and undifferentiated GCs.

## Discussion

*DPYSL3*, located on 5q32 and encoding a 62-kDa protein [11], has been gaining attention as a metastasis modulator [14],[15]. Interestingly, conflicting results have been reported in prostate and pancreatic cancer, implying that *DPYSL3* has a diversity of functions among malignancies. In prostate cancer, the expression of both *DPYSL3* mRNA and protein was inversely associated with lymph node metastasis and VEGF expression, and forced *DPYSL3* expression in cell lines decreased metastasis in a mouse metastatic model [14]. Alternatively, *DPYSL3* promoted adhesion and migration in pancreatic cancer cells in vitro as well as metastasis in vivo via activation of other cell adhesion genes [15]. In this study, the association between *DPYSL3* expression and malignant behavior of GC was investigated.

First, the transcriptional status of *DPYSL3* and potential interacting genes were evaluated in GC cell lines. The expression of *DPYSL3* mRNA was heterogeneous in each GC cell line, and it showed a significant correlation with known tumor promoting factors (*VEGF*, *FAK* and *EZR*) [27]-[29]. These results indicated that *DPYSL3* may be associated with the activation of cancer cell proliferation and metastasis, as is the case with pancreatic cancer. Following the experiments with GC cell lines, an expression analysis using surgical gastric tissues was conducted and provided important findings. *DPYSL3* expression levels in patients with distant metastasis (stage IV) were significantly elevated compared with patients with localized GC (stage I-III), implying that *DPYSL3* upregulation was an important determinant step in the GC progression. Consequently, high expression level of *DPYSL3* mRNA in GC tissues was strongly associated with shortened survival and was identified as an independent prognostic factor. These results indicated that *DPYSL3* upregulation may contribute to GC progression rather than carcinogenesis. Because *DPYSL3* has been reported to play a role in cell adhesion and be a metastatic modulator, the correlations between expression status of *DPYSL3* and metastasis were analyzed. Patients with upregulated *DPYSL3* had a significantly higher prevalence of lymph node metastasis, overall distant metastasis and peritoneal dissemination, indicating that *DPYSL3* is a metastasis facilitator of GC, and high expression of *DPYSL3* may predict the metastatic behavior associated with an invasive GC phenotype. Data from the expression analysis of interacting genes also support the hypothesis that *DPYSL3* has an oncogenic function in GC as with pancreatic cancer [15].

Because GC is considered a biologically heterogeneous disease, and genetic backgrounds can differ according to GC subtype [30]-[33], a subgroup analysis was conducted. Expression status of *DPYSL3* was similar across tumor location (entire, upper third, middle third and lower third). In addition, patients with high expression level of *DPYSL3* mRNA in GCs tended to have a shorter survival both in patient groups of differentiated and undifferentiated GCs. These findings indicated that *DPYSL3* acts similarly in all types of GC.

Although further investigation will be necessary to clarify the underlying molecular mechanism that connects *DPYSL3* upregulation directly to malignant behavior, our findings may offer valuable insight for the specific management of GC patients. Taken together, *DPYSL3* can be used in clinical practice as follows: 1) *DPYSL3* expression levels in the biopsy tissue obtained using endoscopic surveillance samples may identify patients in need of intensive systemic treatment; and 2) *DPYSL3* expression levels in the surgical specimen may be useful for the prediction of an adverse prognosis, also aiding in determining an appropriate therapeutic strategy.

## Conclusion

*DPYSL3* acts as a facilitator of malignant behavior of GC. High expression level of *DPYSL3* in GC tissues may represent a promising biomarker for the malignant behavior of GC.

## Competing interests

The authors declare that they have no competing interests.

## Authors’ contributions

MK, HO, SH, DS, HT and RH performed experiments and data analysis. DK, CT, SY, TF, GN, HS, MK, MF and YK collected cases and clinical data. MK and SN conceived and designed the study, and prepared the initial manuscript. YK supervised the project. All authors contributed to the final manuscript. All authors read and approved the final manuscript.

## Additional file

## Supplementary Material

Additional file 1: Table S1. Primers and annealing temperature. Click here for file
