# Supplementary material for: Dihydropyrimidinase-like 3 facilitates malignant behavior of gastric cancer
Source: J Exp Clin Cancer Res. 2014 Aug 6;33(1):66. doi: 10.1186/s13046-014-0066-9 (PMC4431488; doi:10.1186/s13046-014-0066-9)
Supplement: Additional file 1: Table S1. — Primers and annealing temperature. [file s13046-014-0066-9-S1.doc]

| **Gene** |  | **Oligo sequence (5’ - 3’)** | **Product size** | **Annealing temperature** |
| --- | --- | --- | --- | --- |
| ***DPYSL3*** | forward | AGAAGAAGGAGGGAGGGAGC | 110 bp | 60 °C |
|  | reverse | CTCCCTTGATAAGGAGACGG |  |  |
| ***VEGF*** | forward | CTACCTCCACCATGCCAAGT | 104 bp | 62 °C |
|  | reverse | AGCTGCGCTGATAGACATCC |  |  |
| ***EZR*** | forward | GATAGTCGTGTTTTCGGGGA | 91 bp | 60 °C |
|  | reverse | CTCTGCATCCATGGTGGTAA |  |  |
| ***FAK*** | forward | GCCAAAAGGATTTCTAAACCAG | 110 bp | 64 °C |
|  | reverse | CCTGGTCCACTTGATCAGCTA |  |  |
| ***c-SRC*** | forward | CTGACCGCATGGACCGT | 107 bp | 58 °C |
|  | reverse | AAGCCAACCTGTCACTTGGTA |  |  |
| ***GAPDH*** | forward | GAAGGTGAAGGTCGGAGTC | 226 bp | 60 °C |
|  | probe | CAAGCTTCCCGTTCTCAGCC |  |  |
|  | reverse | GAAGATGGTGATGGGATTTC |  |  |

**Table S1. Primers and annealing temperatures**
